# Prevalence and associated factors of undiagnosed hypertension among adults in the Central African Republic

**DOI:** 10.1038/s41598-022-23868-5

**Published:** 2022-11-08

**Authors:** Supa Pengpid, Karl Peltzer

**Affiliations:** 1 Department of Health Education and Behavioral Sciences, Faculty of Public Health, Mahidol University, Bangkok, Thailand; 2 Department of Public Health, Sefako Makgatho Health Sciences University, Pretoria, South Africa; 3 Department of Healthcare Administration, College of Medical and Health Science, Asia University, Taichung, Taiwan; 4 Department of Psychology, University of the Free State, Bloemfontein, South Africa; 5 Department of Psychology, College of Medical and Health Science, Asia University, Taichung, Taiwan

**Keywords:** Cardiology, Diseases

## Abstract

The study aimed to estimate the prevalence and associated factors of undiagnosed hypertension (HTN) among adults in the Central African Republic (CAR). In the cross-sectional 2017 CAR (Bangui and Ombella M'Poko) STEPS survey, 3265 persons aged 25 to 64 years (non-pregnant and with complete blood pressure measurement), responded to an interview, biomedical and physical, including blood pressure, measurements. Undiagnosed HTN was classified as systolic BP ≥ 140 mmHg and/or diastolic BP ≥ 90 mmHg among adults who had never been told by a doctor or other health worker that they had raised blood pressure or hypertension and had not been taking antihypertensive medication. Binary logistic regressions are used to estimate factors associated with undiagnosed HTN. Among those with HTN (N = 1373), the proportion of undiagnosed HTN was 69.8% and 30.2% diagnosed HTN. In the adjusted logistic regression analysis, male sex (AOR: 2.12, 95% CI 1.39–3.23), current tobacco use (AOR: 1.58, 95% CI 1.03–2.42), and high physical activity (AOR: 1.93, 95% CI 1.00–3.71) were positively associated, and age (AOR: 0.75, 95% CI 0.59–0.96), and underweight (AOR: 0.58, 95% CI 0.37–0.90) were inversely associated with undiagnosed HTN. In addition, among men, ever screened for glucose (AOR: 0.07, 95% CI 0.02–0.27) was negatively associated with undiagnosed HTN, and among women, married or cohabiting (AOR: 1.20, 95% CI 1.00–1.44), current heavy drinking (AOR: 1.41, 95% CI 1.04–1.91) were positively associated with undiagnosed HTN. Seven in ten of the adult population with HTN had undiagnosed HTN in CAR. Efforts should be reinforced to screen for HTN in the general population.

## Introduction

Globally, hypertension (HTN) contributes significantly to morbidity and mortality^1^. Based on population surveys, the prevalence of HTN among adults in low- and middle-income countries (LMICs) was 17.5%^2^, and among adults in sub-Saharan Africa, the prevalence of HTN increased from 19.7% in 1990 to 30.8% in 2010^3^. In the Central African Republic (CAR), the prevalence of HTN among men and women (30–79 years) was 39.5% and 42.8%, respectively^4^. Among adults in LMICs with HTN, 60.8% had undiagnosed HTN^2^, and among adults with HTN in sub-Saharan Africa, the median proportion of undiagnosed HTN was 77.5%^5^. In a small study among individuals (≥ 65 years) with HTN from the CAR, 65.5% had undiagnosed HTN^6^.

If HTN remains undiagnosed and untreated, serious health consequences are indicated, including cardiovascular morbidity and mortality^7,8^. Having HTN not diagnosed can be considered as a problem with the use of health services^9^, conceptualized in the behavioural model of health service utilization by Andersen^10^. According to this model, health care utilization can be conceptualized into predisposing factors (demographic characteristics), enabling factors (objective conditions that may facilitate or impede the use of health services), and health services need factors^10^.

Factors associated with undiagnosed HTN in terms of predisposing health service use can include male sex^8,11–14^, and younger age ^8,12–14^. Enabling/disabling factors include lower economic status^8,11,13^, lower education^13,15^, no health care utilization^12,16^, and health risk behaviours, such as low physical activity^17^, and tobacco use^11,18^. Factors inversely associated with undiagnosed HTN in terms of health service need can include obesity^8,11,14^, underweight^13,16^, other chronic diseases^16^, and diabetes^14^.

Identifying the proportion and factors associated with undiagnosed HTN will help design strategies to prevent and manage the burden of HTN. However, we were unable to identify studies in the general adult population on undiagnosed HTN in CAR. Therefore, this study aimed to estimate the prevalence and associated factors of undiagnosed HTN among adults (25–64 years) in CAR.

## Methods

### Study design and participants

We analyzed data from a subnational cross-sectional survey of adults (25–64 years) who participated in the 2017 CAR STEPS survey in Bangui city and the Ombella M'Poko region with complete measurements of blood pressure and nonpregnant (N = 3,265)^19^. Cluster sampling was used to generate representative data for the age group of 25–64 years in Bangui city and the Ombella M'Poko region^20^. In Bangui, the clusters corresponded to neighbourhoods whose total number was 181 in the second degree. Among these neighbourhoods, a certain number was chosen at random for the random selection of subjects to be included in the sample. In Ombella M'Poko, the clusters corresponded to villages (rural areas)/neighbourhoods (urban areas) in the second degree^20^. The total number of neighbourhoods and villages in Ombella Mpoko was 599. Among these villages and neighbourhoods, a certain number was selected at random^20^. Inclusion criteria were male or female sex, living in urban or rural areas and aged 25–64 years on the day of the survey and residing at least 6 months in the city of Bangui and in the prefecture of Ombella M'poko on the date of the survey and providing informed consent to participate in this study^20^. Exclusion criteria were people who did not give their consent to participate in the study, received two unsuccessful visits, were unable to answer questions, aged < 25 and > 64 years, and have stayed less than 6 months in Bangui or Ombella M'poko^20^. Following the STEPS survey procedures, sociobehavioural information was evaluated in Step 1, physical and blood pressure measurement in Step 2, and biochemical measurements to assess blood glucose and cholesterol based on peripheral blood (capillary) collected at the data collection site in Step 3^20^.

The Ethical Review Committee of the CAR Ministry of Health and Population provided ethics approval of the study, and written informed consent was obtained from the study participants. All methods were carried out in accordance with relevant guidelines and regulations (e.g., Declaration of Helsinki).

### Measures

#### Outcome variable

Undiagnosed HTN was classified as “systolic BP ≥ 140 mmHg and/or diastolic BP ≥ 90 mmHg” among adults who said “no” to the question “Have you ever been told by a doctor or other health worker that you have raised blood pressure or hypertension?” and “no” to the question “During the past two weeks, have you been treated for raised blood pressure with drugs (medication) prescribed by a doctor or other health worker?”^11,20–22^. Diagnosed HTN was defined if they answered “yes” to the question “Have you ever been told by a doctor or other health worker that you have raised blood pressure or hypertension?” and/or “yes” to the question “During the past two weeks, have you been treated for raised blood pressure with drugs (medication) prescribed by a doctor or other health worker?”^11,20,21^.

“Prior to taking blood pressure measurements, participants were asked to sit quietly and rest for 15 min with legs uncrossed. Three readings of systolic and diastolic blood pressure were obtained, with participants resting for three minutes between each reading. Of the three blood pressure measurements using the Omron BP apparatus automatic blood pressure monitor; the last two readings following the recommendations of WHO were averaged”^22^.

*Predisposing factors* consisted of marital status, ethnicity, sex and age^20^.

*Enabling factors* consisted of glucose screening, educational level, household income tertile, current tobacco use, heavy alcohol use in the last month (≥ 5 standard units in men and ≥ 4 units in women in one drinking session), and low, moderate and high physical activity (< 600, 600–1500 and > 1500 metabolic equivalents-min/week, respectively) according to the Global Physical Activity Questionnaire^23^. The tertile of household income in the Central African CFA Franc (XAF) in the past week was grouped into low =  < 7000, medium = 7000 to < 21,000, and high = 21,000 XAF; average exchange rate of the XAF to the United States Dollar (USD) in 2017 was 0.0017 USD^24^.

*The need factors* consisted of diabetes, total cholesterol, and measured body mass index (BMI). BMI was classified as “underweight (< 18.5 kg/m^2^), normal weight (18.5–24.9 kg/m^2^), overweight (25.0–29.9 kg/m^2^), and obesity (≥ 30.0 kg/m^2^).”^22^. *Diabetes:* “fasting plasma glucose levels >  = 7.0 mmol/L (≥ 126 mg/dl); or using insulin or oral hypoglycaemic drugs.”^22^. Total cholesterol (TC) levels were classified as normal: < 190 mg/dl, elevated: 190–239 mg/dl, and high: ≥ 240 mg/dl^22^.

#### Data analysis

The sample and variables are described with frequency statistics. The weighted prevalence of undiagnosed HTN was compared between covariates using Chi-square statistics. *T*-tests were used for comparing means between two groups. Simple and multiple binary logistic regressions using the forced entry method estimated factors associated with undiagnosed HTN. Covariates in the regression model consisted of sex, marital status, age, and ethnicity (predisposing factors), glucose screening, education, household income, heavy alcohol use, current tobacco use, and physical activity (enabling/disabling factors) and total cholesterol, diabetes and BMI (need factors). Covariates were selected based on a previous literature review^8,11–18^. Variables significant at *p* < 0.05 in univariable analyses were incorporated in the multivariable regression model. All statistical procedures were calculated with STATA software version 14.0 (Stata Corporation, College Station, TX, USA), taking the multi-stage sampling design and weighting of the data into account. *p*-values < 0.05 were considered significant, and missing values were discarded.

### Ethics approval and consent to participate

The Ethical Review Committee of the CAR Ministry of Health and Population provided ethics approval of the study, and written informed consent was obtained from the study participants. All methods were carried out in accordance with relevant guidelines and regulations (e.g., Declaration of Helsinki).

## Results

### General characteristics of the study cohort

The sample included 1,373 adults (24–69 years) with HTN. The proportion of undiagnosed HTN was 69.8% and 30.2% diagnosed HTN. The prevalence of undiagnosed HTN was higher in younger age groups, among men, those who had never screened for glucose, were currently using tobacco, current heavy drinkers, those with higher levels of physical activity, those with normal weight, normal total cholesterol, those without diabetes, and with higher diastolic blood pressure. Additional general characteristics of the study cohort are shown in Table 1.

**Table 1:** Characteristics and predisposing, enabling/disabling and need factors in a sample of 1373 adults screened for undiagnosed and diagnosed hypertension (HTN) in Central African Republic, 2017 (% are weighted).

Variable	Total	Undiagnosed HTN^a^	Diagnosed HTN	*p*-value^b^
	N (%)	N (%)	N (%)	
**All**	1373	865 (69.8)	508 (30.2)	
**Predisposing factors**				
Age in years (missing cases = #0) 25–34 35–44 45–54 55–64	112 (18.3) 220 (28.6) 398 (31.0) 643 (22.1)	86 (77.0) 157 (74.1) 266 (70.3) 356 (57.7)	26 (23.0) 63 (25.9) 132 (29.7) 287 (42.3)	< 0.001
Sex (#0) Female Male	898 (50.1) 475 (49.9)	516 (62.1) 349 (77.8)	382 (37.9) 126 (22.2)	< 0.001
Marital status (#27) Not married Married/cohabiting	613 (37.5) 733 (62.5)	373 (67.7) 477 (71.3)	240 (32.3) 256 (28.7)	0.093
Ethnicity (#17) Gbaya or Banda Mandia or Ngbaka Bantou Other	516 (34.8) 313 (24.5) 527 (40.7)	321 (67.0) 201 (71.8) 328 (70.0)	195 (33.0) 112 (28.2) 199 (30.0)	0.434
**Enabling/disabling factors**				
Ever glucose measured (#7) No Yes	1316 (97.4) 50 (2.6)	848 (71.0) 11 (23.1)	468 (29.0) 39 (76.9)	< 0.001
Education in years (#20) 0 1–9 ≥ 10	336 (17.4) 614 (43.6) 403 (39.0)	201 (66.1) 388 (69.1) 262 (71.9)	135 (33.9) 226 (30.9) 141 (28.1)	0.340
Household income in XAF (past week) (#38) < 7000 7000 to < 21,000 ≥ 21,000	363 (23.2) 537 (40.4) 435 (36.4)	233 (70.7) 346 (72.4) 264 (66.9)	130 (29.3) 191 (27.6) 171 (33.1	0.251
Current tobacco use (#3) No Yes	1114 (80.6) 256 (19.4)	673 (67.7) 190 (78.8)	441 (32.3) 66 (21.2)	< 0.001
Current heavy drinking (#1) No Yes	979 (65.7) 393 (34.3)	588 (67.4) 276 (74.6)	391 (32.6) 117 (25.4)	0.025
Physical activity (#25) Low Moderate High	296 (16.7) 171 (11.5) 881 (71.8)	154 (55.5) 104 (67.3) 593 (73.8)	142 (44.5) 67 (32.7) 288 (26.2)	< 0.001
**Need factors**				
Body mass index (#3) Normal Underweight Overweight Obesity	670 (49.3) 192 (14.0) 265 (19.0) 243 (17.7)	438 (73.0) 126 (68.6) 160 (70.4) 138 (61.6)	232 (27.0) 66 (31.4) 105 (29.6) 105 (38.4)	0.041
Diabetes (#346) No Yes	873 (84.8) 154 (15.2)	533 (69.1) 87 (58.4)	340 (30.9) 67 (41.6)	0.025
Total cholesterol (#361) Normal Elevated High	646 (67.7) 258 (23.2) 114 (9.1)	407 (70.4) 141 (59.8) 60 (59.7)	239 (29.6) 117 (40.2) 54 (40.3)	0.016
Blood pressure	M (SD)	M (SD	M (SD)	p-value^c^
Systolic blood pressure	149 (22.7)	148.5 (18.5)	150.5 (30.3)	0.212
Diastolic blood pressure	95.5 (13.7)	96.2 (11.2)	93.9 (18.1)	0.012

*XAF* Central African CFA Franc, Average exchange rate in 2017: 0.0017 USD; ^a^Undiagnosed HTN was classified as systolic BP ≥ 140 mmHg and/or diastolic BP ≥ 90 mmHg among adults who had never been told by a doctor or other health worker that they had raised blood pressure or hypertension and had not been taking antihypertensive medication; ^b^χ^2^ statistic; ^c^*t*-test statistic;

### Associations with undiagnosed hypertension

In adjusted logistic regression analysis, male sex (AOR: 2.12, 95% CI 1.39–3.23), current tobacco use (AOR: 1.58, 95% CI 1.03–2.42), and high physical activity (AOR: 1.93, 95% CI 1.00–3.71) were positively associated, and age (AOR: 0.75, 95% CI 0.59–0.96), and underweight (AOR: 0.58, 95% CI 0.37–0.90) were inversely associated with undiagnosed HTN. In addition, in univariable analysis, ever screened for glucose, obesity, diabetes and elevated total cholesterol were negatively associated with undiagnosed HTN (see Table 2).

**Table 2:** Univariable and multivariable logistic regression reporting unadjusted and adjusted Odds Ratios (OR) for factors associated with undiagnosed hypertension in adults with hypertension in Central African Republic, 2017.

Variable	Unadjusted OR (95% CI)	Adjusted OR (95% CI)^a^
**Predisposing factors**		
Age in years	0.74 (0.61–0.90)**	0.75 (0.59–0.96)*
Sex Female Male	1 (Reference) 2.21 (1.55–3.14)***	1 (Reference) 2.12 (1.39–3.23)**
Marital status Not married Married/cohabiting	1 (Reference) 1.22 (0.89–1.68)	–-
Ethnicity Gbaya or Banda Mandia or Ngbaka Bantou Other	1 (Reference) 1.28 (0.85–1.92) 1.16 (0.72–1.86)	–-
**Enabling/disabling factors**		
Ever glucose measured No Yes	1 (Reference) 0.14 (0.03–0.63)*	1 (Reference) 0.26 (0.03–2.74)
Education in years 0 1–9 ≥ 10	1 (Reference) 1.14 (0.79–1.64) 1.30 (0.55–3.08)	–-
Household income in XAF (past week) < 7000 7000 to < 21,000 ≥ 21,000	1 (Reference) 1.06 (0.71–1.57) 0.79 (0.49–1.28)	–-
Current tobacco use	1.65 (1.17–2.34)**	1.58 (1.03–2.42)*
Current heavy drinking	1.44 (0.84–2.45)	–-
Physical activity Low Moderate High	1 (Reference) 1.76 (1.02–3.03)* 2.46 (1.48–4.10)**	1 (Reference) 1.60 (0.95–2.71) 1.93 (1.00–3.71)*
**Need factors**		
Body mass index Normal Underweight Overweight Obesity	1 (Reference) 0.70 (0.54–0.92)* 0.80 (0.42–1.56) 0.58 (0.35–0.99)*	1 (Reference) 0.58 (0.37–0.90)* 1.01 (0.46–2.31) 0.66 (0.40–1.07)
Diabetes	0.56 (0.32–0.97)*	0.54 (0.21–1.38)
Total cholesterol Normal Elevated High	1 (Reference) 0.66 (0.42–1.04) 0.63 (0.44–0.88)*	1 (Reference) 0.77 (0.52–1.12) 0.87 (0.59–1.28)

*OR* Odds Ratio; *CI* Confidence Intervals; *p < 0.05; **p < 0.01; ***p < 0.001; ^a^Adjusted for age, sex, ever glucose measured, current tobacco use, physical activity, body mass index, diabetes and total cholesterol.

In sex stratified adjusted logistic regression analyses, among men, ever screened for glucose (AOR: 0.07, 95% CI 0.02–0.27) was negatively associated, and high physical activity (AOR: 3.60, 95% CI 1.71–7.58) was positively associated with undiagnosed HTN. In the adjusted logistic regression model among women, married or cohabiting (AOR: 1.20, 95% CI 1.00–1.44), current tobacco use (AOR: 2.08, 95% CI 1.40–3.08), current heavy drinking (AOR: 1.41, 95% CI 1.04–1.91), moderate and high physical activity (AOR: 1.63, 95% CI 1.28–2.08, and AOR: 1.79, 95% CI 1.10–2.91, respectively) were positively associated, and age (AOR: 0.76, 95% CI 0.66–0.88), and underweight (AOR: 0.58, 95% CI 0.40–0.83) were negatively associated with undiagnosed HTN (see Table 3 and 4).

**Table 3:** Univariable and multivariable logistic regression reporting unadjusted and adjusted Odds Ratios (OR) for factors associated with undiagnosed hypertension among male adults with hypertension in Central African Republic, 2017.

Variable	Unadjusted OR (95% CI)	Adjusted OR (95% CI)^a^
**Predisposing factors**		
Age in years	0.74 (0.48–1.13)	–
Marital status Not married Married/cohabiting	1 (Reference) 0.68 (0.29–1.59)	–
Ethnicity Gbaya or Banda Mandia or Ngbaka Bantou Other	1 (Reference) 1.32 (0.92–1.89) 1.18 (0.57–2.45)	–
**Enabling/disabling factors**		
Ever glucose measured No Yes	1 (Reference) 0.05 (0.01–0.21)***	1 (Reference) 0.07 (0.02–0.27)**
Education in years 0 1–9 ≥ 10	1 (Reference) 0.83 (0.56–1.22) 0.76 (0.24–2.44)	–
Household income in XAF (past week) < 7000 7000 to < 21,000 ≥ 21,000	1 (Reference) 0.99 (0.50–1.98) 0.59 (0.29–1.20)	–
Current tobacco use	1.26 (0.50–3.17)	–
Current heavy drinking	1.12 (0.47–2.66)	–
Physical activity Low Moderate High	1 (Reference) 2.25 (0.73–6.93) 3.51 (1.59–7.77)**	1 (Reference) 2.34 (0.77–7.08) 3.60 (1.71–7.58)**
**Need factors**		
Body mass index Normal Underweight Overweight Obesity	1 (Reference) 0.61 (0.36–1.03) 0.53 (0.16–1.72) 0.47 (0.20–1.10)	–
Diabetes	0.50 (0.19–1.30)	
Total cholesterol Normal Elevated High	1 (Reference) 0.54 (0.22–1.29) 0.58 (0.17–2.02)	–

*OR* Odds Ratio; *CI* Confidence Intervals; *p < 0.05; **p < 0.01; ***p < 0.001; ^a^Adjusted for ever glucose measured, and physical activity.

**Table 4:** Univariable and multivariable logistic regression reporting unadjusted and adjusted Odds Ratios (OR) for factors associated with undiagnosed hypertension among female adults with hypertension in Central African Republic, 2017.

Variable	Unadjusted OR (95% CI)	Adjusted OR (95% CI)^a^
**Predisposing factors**		
Age in years	0.70 (0.63–0.79)***	0.76 (0.66–0.88)**
Marital status Not married Married/cohabiting	1 (Reference) 1.43 (1.26–1.61)***	1 (Reference) 1.20 (1.00–1.44)*
Ethnicity Gbaya or Banda Mandia or Ngbaka Bantou Other	1 (Reference) 1.76 (0.79–1.75) 0.98 (0.67–1.45)	–-
**Enabling/disabling factors**		
Ever glucose measured No Yes	1 (Reference) 0.26 (0.03–2.47)	–-
Education in years 0 1–9 ≥ 10	1 (Reference) 1.18 (0.74–1.90) 1.04 (0.44–2.49)	–-
Household income in XAF (past week) < 7000 7000 to < 21,000 ≥ 21,000	1 (Reference) 1.03 (0.69–1.53) 0.75 (0.46–1.24)	–-
Current tobacco use	1.81 (1.29–2.54)**	2.08 (1.40–3.08)**
Current heavy drinking	1.57 (1.11–2.21)*	1.41 (1.04–1.91)*
Physical activity Low Moderate High	1 (Reference) 1.47 (1.15–1.89)** 1.79 (1.21–2.65)**	1 (Reference) 1.63 (1.28–2.08)*** 1.79 (1.10–2.91)*
**Need factors**		
Body mass index Normal Underweight Overweight Obesity	1 (Reference) 0.65 (0.46–0.93)* 1.10 (0.84–1.44) 0.73 (0.49–1.09)	1 (Reference) 0.58 (0.40–0.83)** 1.19 (0.82–1.72) 0.74 (0.49–1.13)
Diabetes	0.62 (0.35–1.08)	–-
Total cholesterol Normal Elevated High	1 (Reference) 0.83 (0.60–1.13) 0.79 (0.56–1.11)	–-

*OR* Odds Ratio; *CI* Confidence Intervals; *p < 0.05; **p < 0.01; ***p < 0.001; ^a^Adjusted for age, current tobacco use, current heavy drinking, physical activity, body mass index, and marital status.

### Discussion

This is the first study to assess the pattern of undiagnosed HTN among adults (25–64 years) in CAR in 2017. The proportion of undiagnosed HTN in CAR (69.8%), was similar to in sub-Saharan Africa (73%)^5^, older adults in CAR (65.5%)^6^, and Peru (67.2%)^14^, higher than in South Africa (49%)^25^, Malaysia (51.6%)^26^, Bangladesh (50.1%)^8^, Nepal (56.9%)^27^, and China (28.8%)^16^, and lower than in Sudan (79.2%)^12^. Some of these country differences can be attributed to less developed health care systems in low-income countries, such as CAR and Sudan, as opposed to more developed health care systems in upper middle-income countries, such as China, Malaysia and South Africa^11,28^. Poor awareness of blood pressure screening can be attributed to the high proportion of undiagnosed HTN in CAR^15^.

We found that older age and underweight decreased the odds of undiagnosed HTN among women, while male sex, current tobacco use, current heavy alcohol use, married and high physical activity increased the odds of undiagnosed HTN among men and/or women. In line with previous findings^8,11–14^, the predisposing factors of male sex and younger age increased the odds of undiagnosed HTN in the current survey. Men have been identified to use health services less often than women, which decreases their opportunity to be tested for blood pressure and diagnosed with HTN^29^. Several studies^12,26^ showed a negative association between being married and undiagnosed HTN, while we found a positive association between being married or cohabiting and undiagnosed HTN among women. This could mean that married or cohabiting women in CAR have less chances to visit health facilities than their single, divorced or widowed counterparts. Programmes geared toward identifying HTN should be aimed at men and the younger women.

Consistent with previous research^11,12,18^, we found that among women current tobacco use, current heavy drinking, and married was associated with undiagnosed HTN, and among men not having been screened for glucose, was associated with undiagnosed HTN. Men who have tested for glucose can use health care services more often and thus reduce undiagnosed HTN^16^. Women who use tobacco and/or are heavy drinkers may be less health conscious about the harmful effects of tobacco use and/or heavy drinking as well as other health concerns, such as hypertension, hindering them to access blood pressure screening. It may be indicated that health awareness and HTN screening is improved among current tobacco users and heavy drinkers, in particular among women, to monitor undiagnosed HTN. Unlike a previous study^17^ that found an association between low physical activity and undiagnosed HTN, we found a positive association between high physical activity and undiagnosed HTN. It is possible that individuals with high physical activity are less health conscious and feel less susceptible to getting chronic diseases^11^. Contrary to several previous studies^8,11,13,15^, we did not find a significant association between education, household income, and undiagnosed HTN. Possible reasons for this nonsignificant effect of socioeconomic status on undiagnosed HTN may be related to the very low Human Development Index (HDI) measured by 'a long and healthy life, access to knowledge and a decent standard of living' of 0.397 in 2019 in CAR (the second lowest of 189 countries and territories)^30^.

According to some research^8,11,14,16^, the health care need factors inversely associated with undiagnosed HTN included obesity, other chronic diseases, and diabetes. Our study showed in univariable analyses a negative association between obesity, diabetes and high total cholesterol with undiagnosed HTN. People without other chronic diseases, such as obesity, diabetes, and high total cholesterol, are less likely to attend health services, thus decreasing their chances of having their blood pressure measured and being diagnosed with HTN. Both (obesity and dyslipidaemia) have been shown to contribute to the development of HTN^31^. Health education is needed to reduce excess body weight and high cholesterol levels^26^.

Overall, health care policy in CAR can increase efforts to screen the general population for HTN together with public awareness campaigns, to reduce the burden of undiagnosed HTN in CAR^6^. Using the WHO Package of Essential Noncommunicable Disease (NCD) Interventions (PEN), the health system must be strengthened to prevent and manage NCD, including HTN^32^.

### Study limitations

The study included only a subnationally representative general population sample in the age range of 25 to 64 years of the CAR, which means that we cannot generalize the findings to the total population of the CAR. The design of the cross-sectional study hinders us in drawing causal conclusions, and some variables were evaluated by self-report, which may have biased responses. Some variables relevant in relation to HTN, such as family history of knowledge of symptoms of HTN, and dietary behaviour, such as sodium consumption, were not measured and should be included in future research. Two of the variables (blood glucose and total cholesterol) assessed had many missing cases (> 25%), which may have contributed to these two variables becoming non-significant in the multivariable analysis.

## Conclusions

Seven in ten adults with HTN had undiagnosed HTN in CAR. Factors identified associated with undiagnosed HTN included younger age, male sex, current tobacco use, high physical activity and not having underweight, and among men, never screened for glucose, and among women, being married or cohabiting and current heavy drinking. Increased HTN screening and public education on HTN are needed to reduce undiagnosed HTN in CAR, and the health system must be strengthened to prevent and manage NCDs, including HTN.

## Data Availability

The data source is publicly available at the World Health Organization NCD Microdata Repository (URL: https://extranet.who.int/ncdsmicrodata/index.php/catalog/737).
